# Gallbladder Polyps Increase the Risk of Ischaemic Heart Disease Among Korean Adults

**DOI:** 10.3389/fmed.2021.693245

**Published:** 2021-08-13

**Authors:** Yong-Jae Lee, Byoungjin Park, Kyung-Won Hong, Dong-Hyuk Jung

**Affiliations:** ^1^Department of Family Medicine, Gangnam Severance Hospital, Seoul, South Korea; ^2^Department of Family Medicine, Yongin Severance Hospital, Yongin, South Korea; ^3^Theragen Bio Co. Ltd., Suwon-si, South Korea

**Keywords:** gallbladder, polyps, coronary disease, comorbidity, cohort study

## Abstract

**Background:** Gallbladder (GB) polyps and ischaemic heart disease (IHD) share some common risk factors. We investigated the longitudinal effects of gallbladder (GB) polyps, as a surrogate metabolic indicator, on IHD.

**Methods:** We enrolled 19,612 participants from the health risk assessment study (HERAS) and Korean Health Insurance Review and Assessment Service (HIRA) database. The primary outcome was IHD, which consisted of angina pectoris (ICD-10 code I20) or acute myocardial infarction (ICD-10 code I21) that occurred after enrolment into the study. We calculated hazard ratios (HRs) with 95% confidence intervals (CIs) for IHD according to the presence of GB polyps using multivariate Cox proportional hazards regression models.

**Results:** The median follow-up period was 29.9 months and a total of 473 individuals (2.4%, 473/19,612) developed IHD. Individuals with GB polyps had an increased risk of IHD compared with the control group after adjusting for potential confounding variables (HR = 1.425; 95% CI, 1.028–1.975). Furthermore, the coexistence of hypertension or dyslipidaemia resulted in an increased risk (HR = 2.14, 95% CI, 1.34–3.44 or HR = 2.09, 95% CI, 1.32–3.31, respectively) of new-onset IHD in the GB polyp group.

**Conclusions:** GB polyps was an independent risk factor of IHD. Awareness of these associations will inform clinicians on the need to include cardiovascular risk management as part of the routine management of patients with GB polyps.

## Introduction

Ischaemic heart disease (IHD) is the leading cause of morbidity and mortality among middle-aged and older individuals globally (1). The incidence of cardiovascular disease (CVD) has increased in developed Asian countries because life style and eating habits have become more westernized (2, 3). Also, IHD carries the 2nd leading death rate following cancer in South Korea, while the recent trend has been gradually rising over the last decade (4). Therefore, it is crucial for physicians to assess the presence of IHD-related risk factors for early prevention of IHD (5).

Gallbladder (GB) polyps are defined based on the presence of polypoidal lesions in the GB mucosa, and ultrasonography (USG) is generally used in clinical settings to detect these polyps (6). USG is a non-invasive tool with >90% sensitivity and specificity for diagnosing GB polyps (7). The prevalence of GB polyps in Korea is estimated at 2.2–8.5%, which is higher than that for Western countries but lower than the prevalence in China (8–10). Although, most GB polyps are cholesterol polyps and benign lesions, the presence of GB polyps is used for the early detection of malignancy and to determine the appropriate time to undergo GB resection surgery (11).

Previous studies suggested that the presence of GB polyps is closely associated with insulin resistance, obesity, and CVD (12, 13). Hence, GB polyps and IHD may share common risk factors; however, few studies have explored the relationship between these two conditions (14).

We conducted a regional and community-based cohort study to investigate the association between GB polyps found by USG and the development of IHD. We included data from the health risk assessment study (HERAS) and Korea Health Insurance Review and Assessment Service (HIRA) database.

## Materials and Methods

### Data and Study Cohort

This retrospective study was derived from the HERAS, which has been previously described in details on design and methodology (15). In brief, the cohort consisted of 20,530 sequential subjects who visited the Health Promotion Center at the Yonsei University Gangnam Severance Hospital for health examinations between November 2006 and June 2010. We excluded 528 participants who had previously been diagnosed with IHD or ischaemic stroke. In addition, patients who met at least one of the following criteria were excluded: <20 years of age, missing data, or high-sensitivity C-reactive protein (hsCRP) levels ≥ 10 mg/L (*n* = 390). Informed consent was obtained from each participant.

Each participant completed a questionnaire that described lifestyle and medical history. The questionnaire on smoking status consists of the following categories: never smoked, current smoker, and former smoker. A regular alcohol drinker was defined as a person who consumed more than 140 g of alcohol per week. Regular exercise was defined as moderate physical activity three or more times per week. Body weight and height were measured to the nearest 0.1 kg and 0.1 cm, respectively, in light indoor clothing without footwear. Body mass index (BMI) was calculated as weight divided by height squared (kg/m^2^). Systolic blood pressure (SBP) and diastolic blood pressure (DBP) were measured in the sitting position after 10 min of rest using a standard mercury sphygmomanometer (Baumanometer, W.A. Baum Co Inc., Copiague, NY, USA). Mean arterial pressure was calculated from SBP and DBP. Dyslipidaemia was defined as total cholesterol ≥ 240 mg/dL, triglycerides ≥ 150 mg/dL, high density-lipoprotein (HDL) cholesterol < 40 mg/dL for men and < 50 mg/dL for women, or the use of lipid-lowering medication. Diabetes was defined as a fasting plasma glucose (FPG) greater or equal to 126 mg/dL, a self-reported history of diabetes, or current use of diabetes medication. Among individuals without diabetes, impaired fasting glucose was defined as FPG levels between 100 and 126 mg/dL.

### Exposures and Outcomes

Experienced radiologists performed abdominal USG using a 3.5-MHz transducer (HDI 5000, Philips, Bothell, WA, USA) and were blinded to laboratory and clinical data. GB polyps were assessed by well-described radiological criteria. A GB polyp was diagnosed if ultrasonography showed any size of hyperechoic mass protruding from the GB wall, and there was no acoustic shadowing or movement with a postural change. The control group without GB polyps consisted of 18,413 participants, and the GB polyp group comprised 1,119 patients. The primary outcome, previously described, was IHD, which consisted of angina pectoris (ICD-10 code I20) or acute myocardial infarction (ICD-10 code I21) that occurred after enrolment into the study (15). To define baseline and post-survey outcomes, we linked a personal 13-digit identification number that was assigned to each participant by the Korean HIRA between November 1, 2006 and December 31, 2010.

### Statistical Analysis

We compared the baseline characteristics according to the presence of GB polyps using Student's *t*-tests for continuous variables and chi-squared tests for categorical variables. We have used box plots and the Kolmogorov-Smirnov test to evaluate the distribution of the variables. Adjusted survival curves were used to estimate the cumulative incidence of IHD for each group. Using the Cox proportional hazards regression model, we calculated hazard ratios (HRs) and 95 % confidence intervals (CIs) for new-onset IHD after adjusting for potential confounding variables. Furthermore, we evaluated whether the presence of GB polyps affected the incidence of IHD when combined with metabolic comorbidities. All analyses were performed using SAS version 9.4 software (SAS Institute Inc., Cary, NC, USA). All statistical tests were two-sided, and statistical significance was set at *P* < 0.05. The data are presented as numbers ± standard deviation or percentage.

## Results

**Figure 1:**
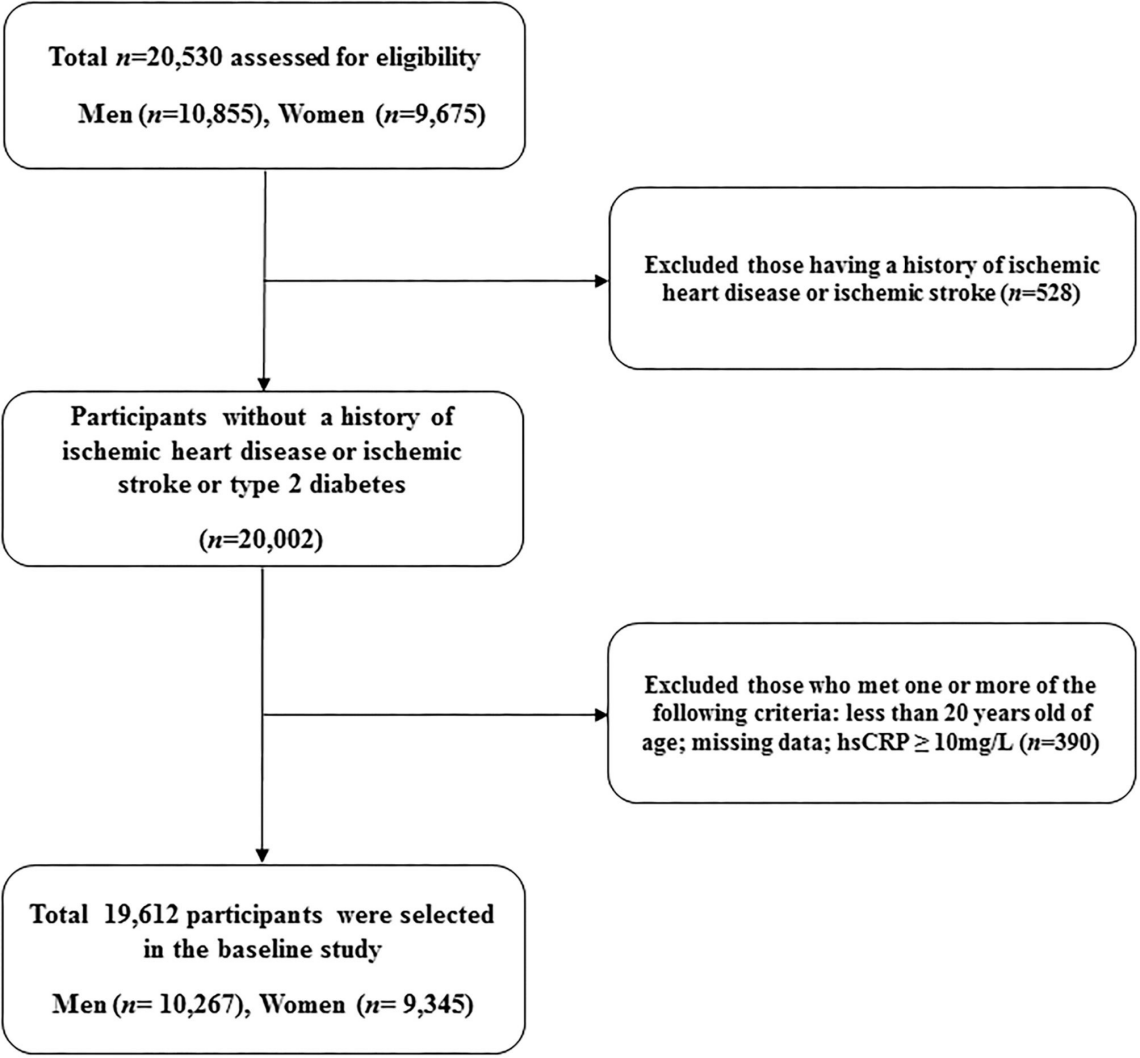
Flowchart for the selection of study participants.

A total of 19,612 participants (10,267 men and 9,345 women) were included in the final analysis (Figure 1). Table 1 presents the baseline characteristics of the HERAS-HIRA cohorts according to the presence of GP polyps. There were no differences in BMI, total cholesterol, FPG, TG, or hsCRP between the two groups. The prevalence of comorbidities, including hypertension, type 2 diabetes mellitus (T2DM), and dyslipidaemia, was not significantly different between the two groups.

**Table 1:** Clinical and biochemical characteristics of the study population.

	**Total (*n* = 19,612)**	**Controls (*n* = 18,413)**	**Gallbladder polyp (*n* = 1,119)**	***P*** **-value^**a**^**
Age, years	45.7 ± 10.9	45.7 ± 10.8	46.8 ± 10.7	<0.001
Male sex, %	52.4	51.9	59.0	<0.001
Body mass index, kg/m^2^	23.4 ± 3.1	23.4 ± 3.1	23.4 ± 3.0	0.939
Systolic blood pressure, mmHg	122.4 ± 15.7	122.4 ± 15.7	122.6 ± 15.5	0.674
Diastolic blood pressure, mmHg	76.3 ± 10.2	76.3 ± 10.2	76.5 ± 10.0	0.668
Mean arterial pressure, mmHg	91.7 ± 11.6	91.7 ± 11.7	91.8 ± 11.4	0.661
Fasting plasma glucose, mg/dL	94.4 ± 18.9	94.3 ± 19.0	95.1 ± 18.4	0.145
Total cholesterol, mg/dL	189.3 ± 34.1	189.3 ± 34.1	190.3 ± 34.2	0.325
Triglyceride, mg/dL	126.3 ± 90.9	126.4 ± 89.8	125.2 ± 106.7	0.715
HDL-cholesterol, mg/dL	53.2 ± 12.7	53.2 ± 12.8	52.0 ± 12.1	<0.001
Aspartate aminotransferase, IU/L	21.9 ± 12.1	21.9 ± 12.3	21.0 ± 8.6	<0.001
Alanine aminotransferase, IU/L	23.3 ± 21.8	23.4 ± 22.1	22.2 ± 15.7	0.015
γ-glutamyltransferase, IU/L	32.3 ± 40.8	32.3 ± 41.3	32.2 ± 31.8	0.894
C-reactive protein, mg/L	1.1 ± 1.4	1.1 ± 1.4	1.0 ± 1.2	0.104
Current smoker, %	24.6	24.6	25.0	0.009
Alcohol drinking, %	43.6	43.8	40.5	0.031
Regular exercise, %	31.7	31.6	33.2	0.270
Hypertension, %	23.2	23.2	24.0	0.495
Type 2 diabetes, %	5.3	5.3	5.1	0.773
Dyslipidaemia, %	38.7	38.8	38.2	0.693
Medication for comorbidities, %				
Hypertension	10.1	10.0	12.3	0.008
Type 2 diabetes	3.0	2.9	3.6	0.176
Dyslipidaemia	3.0	3.0	2.6	0.403
Impaired fasting glucose, %	16.9	16.7	19.9	0.004

a *P-values were calculated using t-test (age, body mass index, systolic blood pressure, diastolic blood pressure, mean arterial pressure, fasting plasma glucose, total cholesterol, triglyceride, HDL-cholesterol, aspartate aminotransferase, alanine aminotransferase, γ-glutamyltransferase, and C-reactive protein) or the chi-squared test (sex, current smoker, alcohol drinking, regular exercise, hypertension, type 2 diabetes, dyslipidaemia, and impaired fasting glucose)*.

*HDL, high density-lipoprotein cholesterol*.

**Figure 2:**
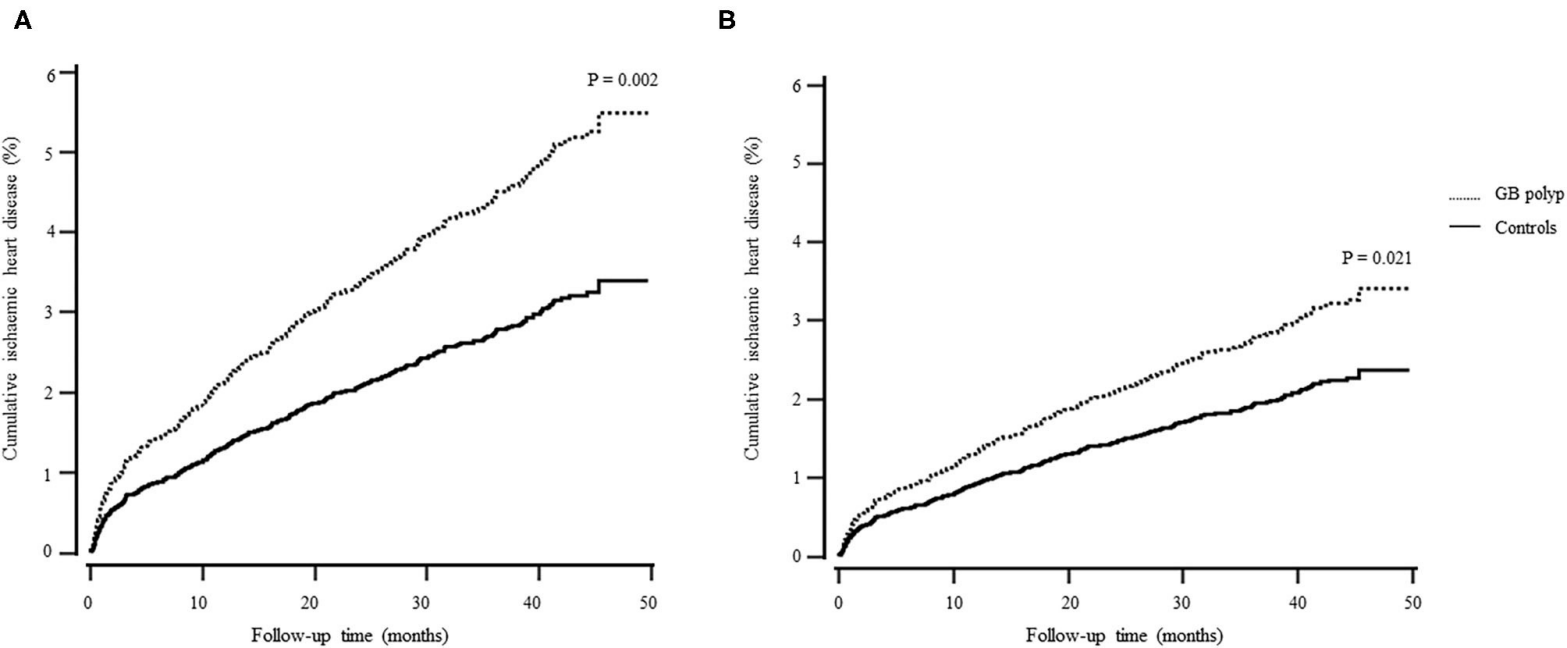
Cox regression survival curve. **(A)** unadjusted; **(B)** adjusted for age, sex, hypertension, diabetes, and dyslipidaemia.

Table 2 shows the incidence of and difference in IHD between the control and GB polyp groups. A total of 473 individuals (2.4%, 473/19,612) developed IHD during the follow-up and the patients with GB polyps had a higher risk of developing IHD. Furthermore, the GB polyp group showed a higher cumulative incidence of IHD over 50 months after adjusting for age, sex, hypertension, diabetes, and dyslipidaemia (*P* = 0.021) (Figure 2).

**Table 2:** Overall incidence of ischaemic heart disease according to the presence of gallbladder polyp.

	**Controls**	**Gallbladder polyp**
New cases of ischaemic heart disease, *n*	430	43
Mean follow-up, years	2.4 ± 1.1	2.2 ± 1.2
Pearson-years of follow-up	43,539	2,613
Incidence rate/1,000 person-years	9.9	16.5

Table 3 presents the univariate and multivariate HRs of IHD categorized by age, sex, alcohol intake, blood tests including lipid profiles, and baseline comorbidities. The increase in IHD risk in the multivariate model was dependent upon greater age, male sex, increased BMI, total cholesterol, or alanine aminotransferase, and GB polyps. Table 4 shows Cox proportional hazard regression analyses for the risk of IHD in patients with GB polyps in the presence of different comorbidities. The coexistence of GB polyps and hypertension or dyslipidaemia resulted in greater risk of IHD compared with GB polyps alone. However, this cumulative effect was observed only in patients with impaired FPG, not in participants with diabetes.

**Table 3:** Univariate and multivariate Cox proportional-hazards regression models for incident ischaemic heart disease.

	**Univariate**			**Multivariate**		
**Variables**	**HRs**	**95% CIs**	***P*** **-value**	**HRs**	**95% CIs**	***P*** **-value**
Age, years	1.071	1.063–1.080	<0.001	1.057	1.047–1.067	<0.001
Male sex, yes or no	1.759	1.453–2.130	<0.001	1.502	1.122–2.010	0.006
Body mass index, kg/m^2^	1.104	1.075–1.135	<0.001	1.046	1.011–1.083	0.010
Current smoking, yes or no	1.264	0.992–1.610	0.058	1.278	0.937–1.741	0.121
Alcohol drinking, yes or no	0.722	0.596–0.876	<0.001	0.766	0.618–0.949	0.014
Regular exercise, yes or no	1.599	1.325–1.930	<0.001	1.163	0.955–1.415	0.132
Mean arterial pressure, mmHg	1.023	1.016–1.031	<0.001	0.996	0.987–1.005	0.354
Fasting plasma glucose, mg/dL	1.011	1.008–1.013	<0.001	1.004	0.999–1.008	0.103
Total cholesterol, mg/dL	1.006	1.003–1.008	<0.001	1.003	1.001–1.006	0.018
Alanine aminotransferase, IU	1.003	1.002–1.004	<0.001	1.004	1.002–1.006	<0.001
C-reactive protein, mg/L	1.116	1.059–1.177	<0.001	1.004	0.939–1.074	0.904
Hypertension medication, yes or no	3.766	3.091–4.588	<0.001	1.714	1.350–2.175	<0.001
Diabetes medication, yes or no	3.163	2.288–4.373	<0.001	1.064	0.715–1.583	0.758
Dyslipidaemia medication, yes or no	4.139	3.103–5.521	<0.001	1.930	1.412–2.638	<0.001
Gallbladder polyp, yes or no	1.632	1.192–2.233	0.002	1.425	1.028–1.975	0.033

**Table 4:** Hazard ratios and 95% confidence intervals for ischaemic heart disease according to gallbladder polyp in the context of metabolic diseases.

	**Model 1**				**Model 2**			
**Comorbidities**	**Controls**		**GB polyp**		**Controls**		**GB polyp**	
	**HRs (95% CIs)**	***P*** **-value**	**HRs (95% CIs)**	***P*** **-value**	**HRs (95% CIs)**	***P*** **-value**	**HRs (95% CIs)**	***P*** **-value**
Hypertension								
No	1.00 (reference)		1.44 (0.94–2.19)	0.090	1.00 (reference)		1.45 (0.93–2.24)	0.100
Yes	1.49 (1.22–1.83)	<0.001	2.14 (1.34–3.44)	0.001	1.52 (1.23–1.88)	<0.001	2.14 (1.32–3.48)	0.002
Type 2 diabetes								
No	1.00 (reference)		1.49 (1.07–2.07)	0.018	1.00 (reference)		1.50 (1.06–2.11)	0.021
Yes	1.46 (1.09–1.94)	0.013	1.59 (0.59–4.26)	0.358	1.50 (1.12–2.02)	0.006	1.58 (0.59–4.23)	0.367
Dyslipidaemia								
No	1.00 (reference)		1.51 (0.98–2.33)	0.059	1.00 (reference)		1.48 (0.94–2.32)	0.089
Yes	1.55 (1.28–1.87)	<0.001	2.09 (1.32–3.31)	0.001	1.54 (1.26–1.88)	<0.001	2.14 (1.33–3.43)	0.001
IFG								
No	1.00 (reference)		1.42 (0.98–2.07)	0.066	1.00 (reference)		1.45 (0.98–2.13)	0.062
Yes	1.35 (1.07–1.70)	0.011	1.90 (1.08–3.32)	0.025	1.41 (1.11–1.80)	0.004	1.87 (1.04–3.35)	0.035

## Discussion

We found that the presence of GB polyps alone was associated with a 42.5% increase in the risk of developing IHD compared with a non-polyp control group. By including IHD risk factors, we discovered that the coexistence of hypertension or dyslipidaemia resulted in an increased risk of IHD in the GB polyp group by approximately 2-fold. However, this cumulative effect was observed only in patients with impaired fasting blood glucose, but not in those with type 2 diabetes mellitus, most of whom are on glucose-lowering medications.

To our knowledge, only one other report has described a relationship between GB polyps and CVD (13). However, in this latter study, the authors did not fully assess IHD-related risk factors to sufficiently show a causal relationship between GB polyps and IHD. In our study, we evaluated multiple risk factors that influence the development of IHD, including blood glucose levels, lipid profiles, blood pressure, liver function, alcohol intake, smoking status, and self-reported diseases, such as T2DM, hypertension, and dyslipidaemia.

It is generally believed that GB disease is positively correlated with metabolic syndrome, obesity, and T2DM because these conditions share common risk factors, such as sedentary life styles, dyslipidaemia, and fat rich diets (12, 16). Our results imply that GB polyps are closely related to the development of IHD, although this association may be an epiphenomenon and not a causal effect. However, our findings suggest that GB polyps may be a risk factor for IHD that is independent of traditional risk factors.

In the present study, patients who presented with GB polyps and the comorbidity hypertension or dyslipidaemia developed an increased risk of IHD that was greater than patients with GB polyps alone. This interaction was observed in patients with impaired FPG, but not in those with T2DM. Considering that the prevalence of metabolic diseases is not different between the GB polyp group and controls at the baseline, both metabolic and non-metabolic factors may contribute to the progression of IHD. Epidemiological studies have reported that the development of IHD is enhanced when CVD risk factors are combined with obesity and metabolic syndrome, but not T2DM, in the general population (17–20). This finding may be explained in part by the medications used (e.g., metformin) and life style modifications that may occur after a diagnosis of T2DM. Metformin has been shown to protect the heart from fibrosis and remodeling after myocardial infarction and decrease inflammation and oxidative stress (21). As a result, patients with T2DM taking metformin may have a weakened risk of developing IHD (22). In addition, our study showed that hypertension had a more obvious effect on IHD development than mean blood pressure at the baseline assessment. This may be related to a follow-up period of <5 years.

Recent epidemiological and experimental studies have reported possible mechanisms by which GB polyps influence the development of IHD. GB polyps are tumor or tumor-like protrusions arising from the GB mucosa and are divided into true polyps and pseudopolyps (23). True polyps are classified as adenomas or adenocarcinomas, and pseudopolyps, which represent over 90% of GB polyps, consist mainly of cholesterol and inflammatory polyps (24, 25). As the names suggest, the growth and development of the majority of GB polyps are closely related with cholesterol metabolism and inflammation (25). Acetyl-CoA acetyltransferase 2 (ACAT2) is a key enzyme in the biogenesis of lipid bodies, which may facilitate the pinocytosis of cholesterol and papillary hyperplasia in the GB mucosa (26). Additionally, this enzyme decreases GB contractility leading to cholesterol deposition in the GB wall (27). ACAT2 is also responsible for incorporation of cholesteryl ester in apoprotein B-containing lipoproteins that leads to increased very low-density lipoprotein (VLDL) secretion and coronary artery atherosclerosis (28). It has been reported that inflammation is closely related to ACAT2 activity and downregulating ACAT2 is associated with lowering cholesterol and preventing atherosclerosis (29). Collectively, the interactive linkage between ACAT activity, inflammation, and dyslipidaemia may have served as a shared mechanism for GB polyps and IHD, an essential starting point for prevention and treatment (30).

Previous cross-sectional studies have consistently found that patients with metabolic syndrome have a high prevalence of GB polyps, suggesting that insulin resistance may be a potential cause (16, 31). An epidemiological study reported that hyperinsulinemia increased the incidence of GB polyp in Korean men (32). Therefore, we propose that screening patients with GB polyps for metabolic disturbances will be important for early detection and prevention of IHD.

The present study had several strengths. First, potential IHD-related confounding factors were assessed by blood tests along with traditional risk factors. Second, the identification of GB polyps occurred during the process of screening the general population rather than relying on the diagnosis by doctors based on symptoms of GB disease. Also, we minimized the misclassification of asymptomatic patients into the control group in our study and decreased cohort bias. USG by experienced radiologists has over 90% of accuracy for the diagnosis of GB polyps (33, 34). Third, this study was carried out using data from a large scale, cohort study to ascertain IHD risk factors and included analyses of multiple physiological tests.

Nevertheless, this study also has some limitations. For the majority of patients with GB polyps, there was no information regarding a pathological condition because the data were obtained from asymptomatic patients who underwent health check-ups. Additionally, this study was a retrospective cohort study and USG was performed only at baseline. Therefore, we were unable to discover new-onset GB polyps during the follow-up period, which may have led to a selection bias. The study cohort was composed of volunteers sequentially visiting for health examination screenings conducted at a single hospital; participants appeared healthier than most community-based cohorts. The number of participants with both GB polyps and comorbidities is small, 1.4% for hypertension, 0.3% for diabetes, and 2.3% for dyslipidaemia. Further research is needed on the risk of IHD according to GB polyps in patients with each cardiometabolic comorbidity. Lastly, individuals with diabetes may have been underestimated because hemoglobin A1c and 2-h oral glucose tolerance tests were not measured at the baseline. Despite these limitations, this is the first study to assess a role for conventional IHD risk factors in the association between GB polyps and development of IHD.

## Conclusions

This study showed that the presence of GB polyps is associated with the development of IHD. Metabolic comorbidities are significant factors that increase the risk of gallbladder polyps associated with IHD. Also, early intervention of GB polyps may reduce IHD risk. These findings may guide the prevention and therapy of patients with both GB polyps and IHD and warrant further studies on effective treatment options.

## Data Availability Statement

The raw data supporting the conclusions of this article will be made available by the authors, without undue reservation.

## Ethics Statement

The studies involving human participants were reviewed and approved by Institutional Review Board of Yonsei University College of Medicine. The patients/participants provided their written informed consent to participate in this study.

## Author Contributions

Y-JL, BP, and D-HJ: study concept and design. Y-JL, BP, and K-WH: acquisition, analysis, and interpretation of data. BP and D-HJ: drafting of the manuscript. Y-JL and D-HJ: critical revision of the manuscript for important intellectual content. All authors contributed to the article and approved the submitted version.

## Conflict of Interest

K-WH was employed by Theragen Bio Co. Ltd. The remaining authors declare that the research was conducted in the absence of any commercial or financial relationships that could be construed as a potential conflict of interest.

## Publisher's Note

All claims expressed in this article are solely those of the authors and do not necessarily represent those of their affiliated organizations, or those of the publisher, the editors and the reviewers. Any product that may be evaluated in this article, or claim that may be made by its manufacturer, is not guaranteed or endorsed by the publisher.

## References

[1] Mendis S Puska P Norrving B Organization WH. Global Atlas on Cardiovascular Disease Prevention and Control. Geneva: World Health Organization (2011).

[2] Hong Y. Burden of cardiovascular disease in Asia: big challenges and ample opportunities for action and making a difference. Clin Chem. (2009) 55:1450–2. 10.1373/clinchem.2009.125369 19498049

[3] Nguyen HN Fujiyoshi A Abbott RD Miura K. Epidemiology of cardiovascular risk factors in Asian countries. Circ J. (2013) 77:2851–9. 10.1253/circj.CJ-13-1292 24240435

[4] Shin H-Y Kim J Lee S Park MS Park S Huh S. Cause-of-death statistics in 2018 in the Republic of Korea. Taehan Uihak Hyophoe Chi. (2020) 63:286–97. 10.5124/jkma.2020.63.5.286

[5] Bansilal S Castellano JM Fuster V. Global burden of CVD: focus on secondary prevention of cardiovascular disease. Int J Cardiol. (2015) 201(Suppl. 1):S1–7. 10.1016/S0167-5273(15)31026-3 26747389

[6] Gallahan WC Conway JD. Diagnosis and management of gallbladder polyps. Gastroenterol Clin North Am. (2010) 39:359–67. 10.1016/j.gtc.2010.02.001 20478491

[7] Andrén-Sandberg A. Diagnosis and management of gallbladder polyps. N Am J Med Sci. (2012) 4:203–11. 10.4103/1947-2714.95897 22655278 PMC3359430

[8] Kim SY Lee HS Lee YS Chung KW Jang BK Chung WJ. Prevalence and risk factors of gallbladder polyp in adults living in Daegu and Gyeongbuk provinces. Korean J Gastroenterol. (2006) 48:344–50. 17132923

[9] Leng S Zhao A Li Q Pei L Zheng W Liang R. Metabolic status and lifestyle factors associated with gallbladder polyps: a covariance structure analysis. BMC Gastroenterol. (2018) 18:159. 10.1186/s12876-018-0882-z 30382815 PMC6211420

[10] Kim HS Cho SK Kim CS Park JS. Big data and analysis of risk factors for gallbladder disease in the young generation of Korea. PLoS ONE. (2019) 14:e0211480. 10.1371/journal.pone.0211480 30794560 PMC6386282

[11] Sun Y Yang Z Lan X Tan H. Neoplastic polyps in gallbladder: a retrospective study to determine risk factors and treatment strategy for gallbladder polyps. Hepatobiliary Surg Nutr. (2019) 8:219–27. 10.21037/hbsn.2018.12.15 31245402 PMC6561872

[12] Chen LY Qiao QH Zhang SC Chen YH Chao GQ Fang LZ. Metabolic syndrome and gallstone disease. World J Gastroenterol. (2012) 18:4215–20. 10.3748/wjg.v18.i31.4215 22919256 PMC3422804

[13] Chen CH Lin CL Kao CH. The risk of coronary heart disease after diagnosis of gallbladder polyp: a retrospective nationwide population-based cohort study. Ann Transl Med. (2019) 7:753. 10.21037/atm.2019.11.114 32042769 PMC6990032

[14] Chen CH Lin CL Kao CH. Association of gallbladder polyp and stroke: a nationwide, population-based study. Medicine (Baltimore). (2015) 94:e2192. 10.1097/MD.0000000000002192 26632906 PMC4674209

[15] Park B Lee YJ Lee HS Jung DH. The triglyceride-glucose index predicts ischemic heart disease risk in Koreans: a prospective study using National Health Insurance Service data. Cardiovasc Diabetol. (2020) 19:210. 10.1186/s12933-020-01186-2 33302952 PMC7731566

[16] Park EJ Lee HS Lee SH Chun HJ Kim SY Choi YK. Association between metabolic syndrome and gallbladder polyps in healthy Korean adults. J Korean Med Sci. (2013) 28:876–80. 10.3346/jkms.2013.28.6.876 23772152 PMC3678004

[17] Després JP. Potential contribution of metformin to the management of cardiovascular disease risk in patients with abdominal obesity, the metabolic syndrome and type 2 diabetes. Diabetes Metab. (2003) 29:6s53–61. 10.1016/S1262-3636(03)72788-8 14502101

[18] Griffin SJ Leaver JK Irving GJ. Impact of metformin on cardiovascular disease: a meta-analysis of randomised trials among people with type 2 diabetes. Diabetologia. (2017) 60:1620–9. 10.1007/s00125-017-4337-9 28770324 PMC5552849

[19] Katsiki N Purrello F Tsioufis C Mikhailidis DP. Cardiovascular disease prevention strategies for type 2 diabetes mellitus. Expert Opin Pharmacother. (2017) 18:1243–60. 10.1080/14656566.2017.1351946 28685623

[20] Lorber D. Importance of cardiovascular disease risk management in patients with type 2 diabetes mellitus. Diabetes Metab Syndr Obes. (2014) 7:169–83. 10.2147/DMSO.S61438 24920930 PMC4043722

[21] Jenkins AJ Welsh P Petrie JR. Metformin, lipids and atherosclerosis prevention. Curr Opin Lipidol. (2018) 29:346–53. 10.1097/MOL.0000000000000532 29878903

[22] Forouzandeh F Salazar G Patrushev N Xiong S Hilenski L Fei B. Metformin beyond diabetes: pleiotropic benefits of metformin in attenuation of atherosclerosis. J Am Heart Assoc. (2014) 3:e001202. 10.1161/JAHA.114.001202 25527624 PMC4338706

[23] Lee KF Wong J Li JC Lai PB. Polypoid lesions of the gallbladder. Am J Surg. (2004) 188:186–90. 10.1016/j.amjsurg.2003.11.043 15249249

[24] Zemour J Marty M Lapuyade B Collet D Chiche L. Gallbladder tumor and pseudotumor: diagnosis and management. J Visc Surg. (2014) 151:289–300. 10.1016/j.jviscsurg.2014.05.003 24930718

[25] Myers RP Shaffer EA Beck PL. Gallbladder polyps: epidemiology, natural history and management. Can J Gastroenterol. (2002) 16:187–94. 10.1155/2002/787598 11930198

[26] Parini P Davis M Lada AT Erickson SK Wright TL Gustafsson U. ACAT2 is localized to hepatocytes and is the major cholesterol-esterifying enzyme in human liver. Circulation. (2004) 110:2017–23. 10.1161/01.CIR.0000143163.76212.0B 15451793

[27] Buhman KK Accad M Novak S Choi RS Wong JS Hamilton RL. Resistance to diet-induced hypercholesterolemia and gallstone formation in ACAT2-deficient mice. Nat Med. (2000) 6:1341–7. 10.1038/82153 11100118

[28] Genoula M Marín Franco JL Dupont M Kviatcovsky D Milillo A Schierloh P. Formation of foamy macrophages by tuberculous pleural effusions is triggered by the interleukin-10/signal transducer and activator of transcription 3 axis through ACAT upregulation. Front Immunol. (2018) 9:459. 10.3389/fimmu.2018.00459 29593722 PMC5854656

[29] Kavanagh K Davis MA Zhang L Wilson MD Register TC Adams MR. Estrogen decreases atherosclerosis in part by reducing hepatic acyl-CoA:cholesterol acyltransferase 2 (ACAT2) in monkeys. Arterioscler Thromb Vasc Biol. (2009) 29:1471–7. 10.1161/ATVBAHA.109.191825 19759374 PMC2763273

[30] Watanabe F Hanai H Kaneko E. Increased acylCoA-cholesterol ester acyltransferase activity in gallbladder mucosa in patients with gallbladder cholesterolosis. Am J Gastroenterol. (1998) 93:1518–23. 10.1111/j.1572-0241.1998.00473.x 9732935

[31] Dilek ON Karasu S Dilek FH. Diagnosis and treatment of gallbladder polyps: current perspectives. Euroasian J Hepatogastroenterol. (2019) 9:40–8. 10.5005/jp-journals-10018-1294 31988866 PMC6969319

[32] Chang Y Sung E Ryu S Park YW Jang YM Park M. Insulin resistance is associated with gallstones even in non-obese, non-diabetic Korean men. J Korean Med Sci. (2008) 23:644–50. 10.3346/jkms.2008.23.4.644 18756051 PMC2526403

[33] Martin E Gill R Debru E. Diagnostic accuracy of transabdominal ultrasonography for gallbladder polyps: systematic review. Can J Surg. (2018) 61:200. 10.1503/cjs.011617 29806818 PMC5973908

[34] Mellnick VM Menias CO Sandrasegaran K Hara AK Kielar AZ Brunt EM. Polypoid lesions of the gallbladder: disease spectrum with pathologic correlation. Radiographics. (2015) 35:387–99. 10.1148/rg.352140095 25763724

